# Maternal Urinary Iodine Concentration during Pregnancy and Its Impact on Child Growth and Neurodevelopment: An 11-Year Follow-Up Study

**DOI:** 10.3390/nu15204447

**Published:** 2023-10-20

**Authors:** Carla A. Lopes, Marta Duarte, Susana Prazeres, Ivone Carvalho, Laura Vilarinho, José Martinez-de-Oliveira, Edward Limbert, Manuel C. Lemos

**Affiliations:** 1 CICS-UBI, Health Sciences Research Centre, University of Beira Interior, 6200-506 Covilhã, Portugal; ccasantos5@gmail.com (C.A.L.); martad@fcsaude.ubi.pt (M.D.); jmo@fcsaude.ubi.pt (J.M.-d.-O.); 2 Departamento da Saúde da Criança e da Mulher, Centro Hospitalar Universitário Cova da Beira, 6200-251 Covilhã, Portugal; 3 Laboratório de Endocrinologia, Serviço de Patologia Clínica, Instituto Português de Oncologia de Lisboa Francisco Gentil, 1099-023 Lisboa, Portugal; sprazeres@ipolisboa.min-saude.pt; 4 Unidade de Rastreio Neonatal, Metabolismo e Genética, Departamento de Genética Humana, Instituto Nacional de Saúde Doutor Ricardo Jorge, 4000-055 Porto, Portugal; ivone.carvalho@insa.min-saude.pt (I.C.); laura.vilarinho@insa.min-saude.pt (L.V.); 5 Serviço de Endocrinologia, Instituto Português de Oncologia de Lisboa Francisco Gentil, 1099-023 Lisboa, Portugal; edwardlimbert@gmail.com

**Keywords:** iodine, thyroid, pregnancy, growth, IQ, neurodevelopment

## Abstract

Mild-to-moderate iodine deficiency during pregnancy is prevalent worldwide, but its consequences for maternal and child health are not clear. We aimed to investigate the impact of maternal iodine intake during pregnancy on the child’s growth and neurodevelopment. This study involved a cohort of 11-year-old children (*n* = 70) whose mothers had participated in an iodine intake survey during pregnancy. Gestational, neonatal, anthropometric, intelligence quotient (IQ), and socioeconomic parameters were analyzed according to maternal urinary iodine concentration (UIC). There was a positive linear trend of current height Z-score, full-scale IQ, verbal IQ, family income, maternal education, and a negative trend of neonatal TSH levels with increasing maternal UIC levels. However, regression analysis indicated that maternal UIC was not an independent predictor of any gestational, neonatal, or childhood development parameter. Only maternal school education was positively associated with child height and IQ. In conclusion, we did not find any evidence of a direct effect of maternal iodine intake during pregnancy on the long-term growth and neurodevelopment of children. The results suggest that socioeconomic factors are important confounding factors that affect both maternal iodine intake and child development and must be considered when investigating the association between maternal iodine intake and child outcomes.

## 1. Introduction

Iodine is an essential micronutrient required for the biosynthesis of thyroid hormones, which play a crucial role in the regulation of metabolism, growth, and development [1]. An adequate dietary iodine intake during pregnancy and early childhood is critically important for growth and neurodevelopment [2].

Iodine deficiency remains a public health issue in many parts of the world [3]. As most ingested iodine is excreted in the urine, the median urinary iodine concentration (UIC) can be used to assess iodine nutrition in a population. According to the World Health Organization (WHO), a median UIC lower than 100 µg/L (or 150 µg/L in pregnant women) indicates an insufficient iodine intake [4].

Pregnant women are particularly susceptible to iodine deficiency due to increased thyroid hormone production, increased renal iodine excretion, and fetal iodine requirements [5]. Severe maternal iodine deficiency, particularly during the first trimester of pregnancy, can lead to negative outcomes, such as intellectual impairment, adverse obstetric outcomes, and cretinism [6]. Improved nutrition and universal salt iodization have been shown to prevent these effects in severely iodine-deficient regions [7,8].

Although severe iodine deficiency has been significantly reduced worldwide, mild-to-moderate iodine deficiency still remains prevalent in many countries, including in Europe and North America [3]. In contrast to severe iodine deficiency, the consequences of mild-to-moderate iodine deficiency for maternal and child health are still unclear. Lower UIC values in pregnancy have been associated with lower working memory in a Dutch study cohort of 4-year-old children [9], lower verbal intelligence in a British study cohort of 8-year-olds [10], lower language and spelling scores in an Australian study cohort of 9- and 15-year-olds [11,12], lower cognitive scores in a Spanish study cohort of 5-year-olds [13], and lower language skills in a Norwegian study cohort of 18-month-olds [14]. In contrast, no associations with maternal UIC were found in a Spanish study cohort of 12- to 30-month-olds [15,16], a Dutch study cohort of 6-year-olds [17], a Norwegian study cohort of 8-year-olds [18], an Australian study cohort of 18-month-olds [19], a British study cohort of 4- to 7-year-olds [20], or a Bangladeshi study cohort of 5- to 10-year-olds [21]. In addition, iodine supplementation trials in pregnancy have mostly failed to demonstrate a beneficial effect on child development in regions of mild-to-moderate iodine deficiency [22,23]. The inconsistency in results may be attributed to differences in methods for assessing iodine and child development, severity of the iodine deficiency in the population, and adjustment for potential confounding variables [24]. Given these contradictory data, the real effects of maternal mild-to-moderate iodine deficiency on child outcomes remain to be clarified.

The aim of this study was to investigate the impact of urinary iodine concentration during pregnancy on the growth and neurodevelopment of 11-year-old Portuguese children.

## 2. Materials and Methods

### 2.1. Subjects

**Figure 1:**
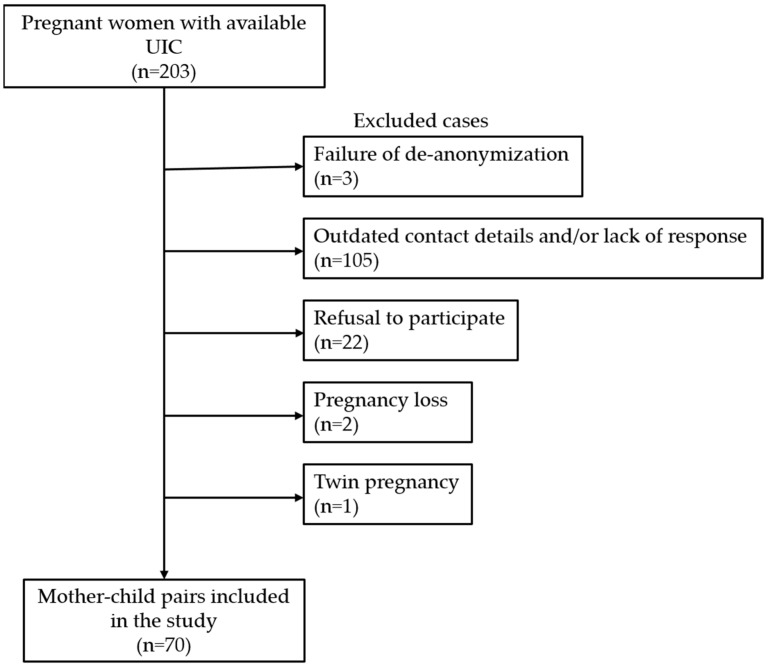
Flowchart of the study design. UIC, urinary iodine concentration.

This was a follow-up study of an iodine intake survey in Portuguese pregnant women published in 2010 [25]. We used the data of 203 pregnant women that had been recruited at a hospital-based outpatient clinic (Centro Hospitalar Universitário Cova da Beira, Covilhã, Portugal) while undertaking routine pregnancy check-ups and screening tests. This regional hospital is located in the hinterland of continental Portugal, which is historically known as an iodine-deficient region [25,26,27]. None of these women had taken iodine supplements during pregnancy at currently recommended levels, as national guidelines promoting iodine supplementation during pregnancy were only established in 2013 [28]. Iodine status in these pregnant women had been assessed by determining the UIC in morning spot urine samples using a fast colorimetric method appropriate for population studies [29]. The median UIC in this group was 67.6 µg/L and the mean gestational age at measurement ± standard deviation (SD) was 29.0 ± 9.9 weeks [25]. As data from the previous study [25] had been anonymized (urine samples and results had been encoded with the initials of the woman’s name, gestational age, and date of urine collection), de-anonymization of the participants was carried out by cross-referencing the encoded identifiers to pregnant women attending the hospital during the relevant period. Only women that were unambiguously identified were selected for the next step. We searched the records of these women to retrieve their contact details. Enrolment began by sending a written invitation to their home address followed by a telephone call. Then, an appointment was arranged, at which point informed consent was requested and, if consent was granted, the child underwent further assessment. Of the original 203 women, 133 were excluded from this study due to failure of de-anonymization (*n* = 3), outdated contact details and/or lack of response (*n* = 105), refusal to participate (*n* = 22), pregnancy loss (*n* = 2), and twin pregnancy (*n* = 1) (Figure 1). Thus, a total of 70 mother–child pairs were included in this study (39 boys, 31 girls, mean age ± SD = 11.3 ± 0.7 years). This study was approved by the Institutional Ethics Committee of the Centro Hospitalar Universitário Cova da Beira, Covilhã (Ref: 35/2017).

### 2.2. Pregnancy and Neonatal Parameters

Pregnancy and birth outcome data were collected through a review of medical records. Recorded data included UIC during pregnancy; gestational age at UIC measurement; gestational age at birth; and length, weight, and head circumference at birth. Neonatal thyroid-stimulating hormone (TSH) levels determined with routine Guthrie tests were retrieved from the National Neonatal Screening Program (Instituto Nacional de Saúde Doutor Ricardo Jorge, Porto, Portugal).

### 2.3. Socioeconomic Parameters

Each mother was asked to provide information on family income (measured in multiples of the national minimum wage) and the number of years of maternal school education.

### 2.4. Assessment of Child Development

Each child was assessed by a certified nurse (the author C.A.L.) and psychologist (the author M.D.), who were blinded for the mother’s iodine intake in pregnancy. Height and weight were measured and converted into age- and sex-adjusted height and body mass index (BMI) Z-scores. The child then undertook a WISC-III test (Wechsler Intelligence Scale for Children, 3rd ed., Portuguese edition, 2003, Hogrefe Ltd., Lisboa, Portugal) to determine the verbal, performance, and full-scale intelligence quotients (IQs). In 18 children, an IQ test had already been performed for different purposes and, to avoid repeating the test, these results were used instead.

### 2.5. Statistical Analysis

Maternal UICs (µg/L) were assigned to categories of an ordinal variable, as previously described [25]. Children were divided into five groups according to maternal UIC (<25, 25–50, 50–100, 100–150, and >150 µg/L). These categories of UIC were based on those used by the WHO for population studies of iodine nutrition [4]. Neonatal, socioeconomic, and child development parameters for each group are expressed as means ± SD and compared between the groups via one-way analysis of variance (ANOVA) with a post-test for linear trend (GraphPad Prism, Version 7.04 for Windows, GraphPad Software, San Diego, CA, USA). To account for the effect of possible confounding variables on child development, multiple linear regression was performed using each child development parameter as the dependent variable. A stepwise variable selection method with an F probability of entry and removal of 0.05 and 0.10, respectively, was carried out, along with pairwise exclusion of missing values (missing values: length at birth, 14 cases; head circumference, 13 cases; TSH, 1 case; family income, 1 case; and maternal school education, 1 case) (SPSS Statistics, Version 28.0.0 for Windows, IBM, Armonk, NY, USA).

## 3. Results

**Figure 2:**
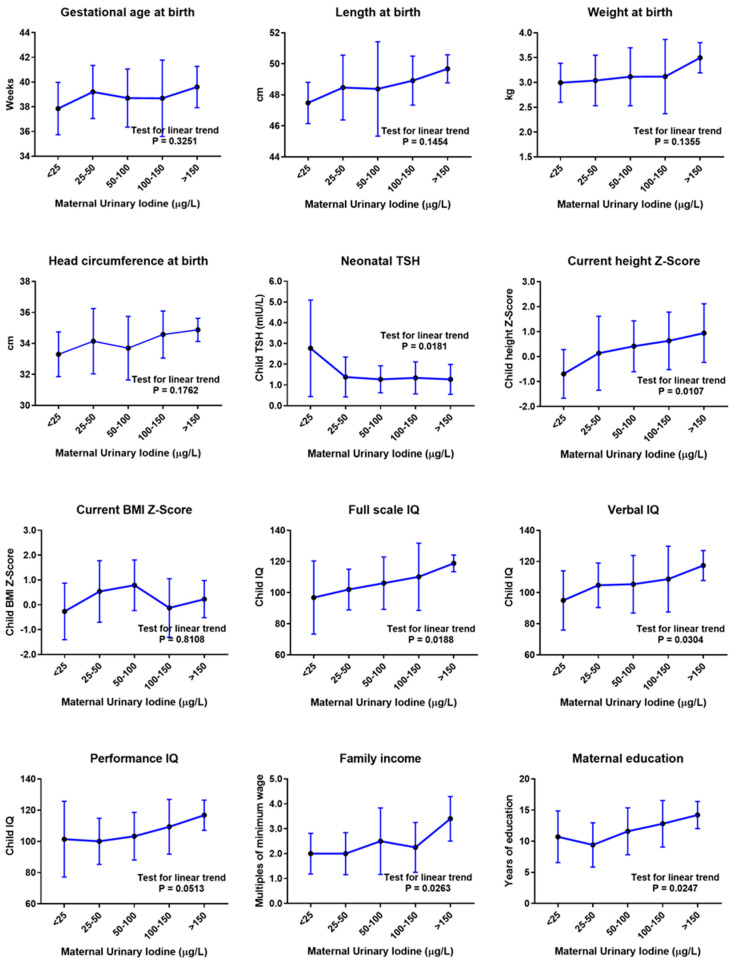
Child outcomes according to maternal urinary iodine concentration (UIC) in pregnancy, before adjustment for confounding variables. Mean values and standard deviation error bars are presented for each UIC category. Mean values were compared via one-way analysis of variance (ANOVA) with post-test for linear trend. µg/L, micrograms per liter; cm, centimeters; kg, kilograms; TSH, thyroid-stimulating hormone; mIU/L, milli-international units per liter; BMI, body mass index; IQ, intelligence quotient.

The gestational, neonatal, current physical and neurodevelopment, and socioeconomic parameters of the 70 children, according to maternal UIC in pregnancy, are presented in Table 1. ANOVA and post-test analysis revealed a positive linear trend of current height Z-score (*p* = 0.0107), verbal IQ (*p* = 0.0304), full-scale IQ (*p* = 0.0188), family income (*p* = 0.0263), and maternal education (*p* = 0.0247) and a negative linear trend of neonatal TSH levels (*p* = 0.0181) with increasing maternal UIC levels (Figure 2).

However, linear regression analysis showed that maternal UIC was not an independent predictor for any gestational, neonatal, or childhood development parameter (Table 2). In particular, maternal UIC was not associated with current height Z-score (standardized coefficient beta (β) = 0.185, *p* = 0.167), verbal IQ (β = 0.135, *p* = 0.319), performance IQ (β = 0.156, *p* = 0.241), full-scale IQ (β = 0.173, *p* = 0.187), or neonatal TSH (β = −0.175, *p* = 0.212).

Interestingly, linear regression analysis, using child IQ as the dependent variable, revealed that only maternal school education was positively associated with child IQ. The number of years of maternal school education was associated with child full-scale IQ (regression coefficient (β) = 0.430, *p* = 0.001), verbal IQ (β = 0.383, *p* = 0.004) and performance IQ (β = 0.407, *p* = 0.002). Additionally, maternal education was independently associated with length at birth (β = 0.287, *p* = 0.033) and current height Z-score (β = 0.394, *p* = 0.003) and negatively associated with neonatal TSH (β = −0.280, *p* = 0.038). Importantly, maternal education was independently associated with maternal UIC (β = 0.317, *p* = 0.018) (Table 2).

## 4. Discussion

This study involved a cohort of 11-year-old children whose mothers had been studied for iodine intake during pregnancy [25]. Our study did not demonstrate a significant effect of pregnancy iodine intake on any gestational, neonatal, or childhood development parameter. In particular, growth and neurocognitive development at the age of 11 years were not dependent on maternal UIC during pregnancy.

Although pregnancy UIC was apparently associated with child height and IQ, regression analysis showed that this was an indirect association due to the effect of maternal education on these variables. Indeed, the number of years of maternal school education was the only independent predictor of child height and IQ. This suggests that maternal education and other socioeconomic variables are important confounding variables that must be considered when interpreting studies on the outcome of maternal iodine intake. In this study, failure to adjust for these variables could have easily resulted in an erroneous conclusion of a causal relationship between maternal UIC and child development.

The association of child growth and IQ with socioeconomic factors is not surprising. Socioeconomic status has been associated with the performance of children on intelligence tests [30]. Children of the highest and lowest socioeconomic status backgrounds have been shown to be on average separated by up to 17 IQ points [30]. Children from less-educated mothers have also been shown to be shorter on average [31]. Additionally, socioeconomic factors can influence dietary intake, including iodine intake during pregnancy [32], which may explain the observed association between maternal education and maternal UIC. Therefore, our results suggest that maternal UIC is a surrogate marker of maternal education, and that the latter is the real predictor of child development.

We also found an inverse association between maternal education and child neonatal TSH level. As TSH is secreted by the pituitary in response to low levels of thyroid hormones, an increase in neonatal TSH has been suggested to be a good indicator of iodine deficiency [4]. Therefore, it is not surprising that an inverse association with neonatal TSH also accompanied the observed association between maternal education and maternal UIC.

Mild-to-moderate iodine deficiency during pregnancy has been associated with adverse child neurodevelopmental outcomes in some [9,10,11,12,13,14] but not all studies [15,16,17,18,19,20,21]. In addition, intervention trials have not convincingly shown a beneficial effect of iodine supplementation during pregnancy on child development in regions of mild-to-moderate iodine deficiency [22,23]. These discrepancies may be related to differences in the studied populations or in the methods used to assess iodine status or child development, including available data on confounders [24]. A recent study showed that maternal iodine deficiency was associated with lower full-scale and verbal scores at 5 and 10 years, but this association disappeared after adjusting for family socioeconomic status and maternal education [21]. Along with our findings, this suggests that mild-to-moderate maternal iodine deficiency has a relatively small effect on long-term child development and other influences, such as child diet and environmental and socioeconomic factors, may outweigh any suboptimal iodine exposure during pregnancy.

Despite the lack of high-quality evidence, several scientific societies have recommended iodine supplementation during pregnancy in areas of mild-to-moderate iodine deficiency [33,34,35]. However, the impact of these recommendations on child outcomes remains to be determined.

A strength of our study is that it is one of the longest (over 11 years) follow-up studies of children born to mothers with known iodine intake. Another strength is that this study was carried out in an iodine deficient region and maternal iodine intake was assessed before iodine supplementation in pregnancy became routine practice, allowing the investigation of outcomes over a wide range of iodine deficiency. As iodine supplementation in pregnancy becomes more widespread, it will become increasingly difficult to undertake similar studies.

A limitation of our study is the relatively small sample size. From the original cohort of 203 pregnant women [25], only 70 mother–child pairs were available for the current study, mostly due to difficulties in re-establishing contacts with the mothers more than 11 years since the original iodine intake survey. Also, there were limitations in assessing iodine status from single spot urine samples. Although our measurements of UIC followed WHO recommendations for population surveys, individual UIC values may vary from day to day, depending on recent iodine intake, and may not reflect the usual iodine status for that individual. Other studies have suggested that dietary habits may be a more sensitive indicator of iodine deficiency than urinary iodine [36].

## 5. Conclusions

This study did not show any significant impact of maternal iodine intake during pregnancy on the long-term growth and neurocognitive development of children. Socioeconomic factors are confounding factors that affect both maternal iodine intake and child development and must be taken into account when interpreting studies on maternal iodine intake and child outcomes. Further research is needed to determine the impact of iodine intake during pregnancy on child outcomes in areas of mild-to-moderate iodine deficiency.

## Figures and Tables

**Table 1:** Characteristics of children according to maternal urinary iodine concentration in pregnancy.

	Maternal UIC (µg/L)					
	All	<25	25–50	50–100	100–150	>150
Number, n	70	7	15	27	16	5
Sex (M/F), n	39/31	5/2	8/7	13/14	10/6	3/2
Gestational age at iodine status (weeks), mean ± SD	29.6 ± 9.3	27.0 ± 9.2	30.9 ± 9.6	27.2 ± 9.5	31.5 ± 9.7	35.0 ± 2.6
Gestational age at birth (weeks), mean ± SD	38.8 ± 2.4	37.9 ± 2.1	39.2 ± 2.1	38.7 ± 2.4	38.7 ± 3.1	39.6 ± 1.7
Length at birth (cm), mean ± SD	48.5 ± 2.3	47.5 ± 1.3	48.5 ± 2.1	48.4 ± 3.0	48.9 ± 1.6	49.7 ± 0.9
Weight at birth (kg), mean ± SD	3.11 ± 0.58	2.99 ± 0.39	3.04 ± 0.51	3.12 ± 0.58	3.12 ± 0.75	3.50 ± 0.30
Head circumference at birth (cm), mean ± SD	34.0 ± 1.9	33.3 ± 1.4	34.1 ± 2.1	33.7 ± 2.1	34.6 ± 1.5	34.9 ± 0.8
Neonatal TSH (mIU/L), mean ± SD	1.46 ± 1.09	2.77 ± 2.33	1.39 ± 0.96	1.27 ± 0.62	1.34 ± 0.77	1.27 ± 0.72
Age at IQ evaluation (years), mean ± SD	11.3 ± 0.7	11.4 ± 0.9	11.3 ± 0.8	11.3 ± 0.8	11.1 ± 0.7	10.9 ± 0.2
Current height Z-score, mean ± SD	0.33 ± 1.21	−0.69 ± 0.98	0.13 ± 1.48	0.41 ± 1.02	0.63 ± 1.15	0.94 ± 1.17
Current BMI Z-score, mean ± SD	0.38 ± 1.15	−0.27 ± 1.14	0.54 ± 1.24	0.79 ± 1.02	−0.12 ± 1.18	0.23 ± 0.75
Verbal IQ, mean ± SD	105.8 ± 18.1	95.0 ± 19.0	104.7 ± 14.3	105.4 ± 18.5	108.7 ± 21.1	117.4 ± 9.6
Performance IQ, mean ± SD	104.8 ± 16.7	101.4 ± 24.3	100.1 ± 14.7	103.3 ± 15.3	109.4 ± 17.5	116.8 ± 9.7
Full-scale IQ, mean ± SD	106.1 ± 17.9	96.9 ± 23.5	102.0 ± 13.1	106.1 ± 16.9	110.2 ± 21.6	118.8 ± 5.4
Family income (multiples of minimum wage), mean ± SD	2.3 ± 1.1	2.0 ± 0.8	2.0 ± 0.9	2.5 ± 1.3	2.3 ± 1.0	3.4 ± 0.9
Maternal education (years), mean ± SD	11.6 ± 3.8	10.7 ± 4.2	9.4 ± 3.6	11.6 ± 3.8	12.8 ± 3.7	14.2 ± 2.4

UIC, urinary iodine concentration; µg/L, microgram per liter; n, number; M, male; F, female; SD, standard deviation; cm, centimeters; kg, kilograms; TSH, thyroid-stimulating hormone; mIU/L, milli-international units per liter; IQ, intelligence quotient; BMI, body mass index.

**Table 2:** Linear regression analysis of gestational, neonatal, and childhood development parameters.

	Independent Variables	
	Maternal UIC	Maternal Education
Maternal UIC	-	β = 0.317 *p* = 0.018 *
Gestational age at birth	β = −0.046 *p* = 0.649	β = 0.004 *p* = 0.967
Length at birth	β = 0.112 *p* = 0.424	β = 0.287 *p* = 0.033 *
Weight at birth	β = 0.035 *p* = 0.718	β = −0.062 *p* = 0.528
Head circumference at birth	β = 0.121 *p* = 0.217	β = 0.040 *p* = 0.687
Neonatal TSH	β = −0.175 *p* = 0.212	β = −0.280 *p* = 0.038 *
Current height Z-score	β = 0.185 *p* = 0.167	β = 0.394 *p* = 0.003 *
Current BMI Z-score	β = −0.132 *p* = 0.344	β = −0.195 *p* = 0.176
Verbal IQ	β = 0.135 *p* = 0.319	β = 0.383 *p* = 0.004 *
Performance IQ	β = 0.156 *p* = 0.241	β = 0.407 *p* = 0.002 *
Full-scale IQ	β = 0.173 *p* = 0.187	β = 0.430 *p* = 0.001 *
Family income	β = 0.105 *p* = 0.413	β = 0.478 *p* < 0.001 *
Maternal education	β = 0.212 *p* = 0.089	-

UIC, urinary iodine concentration; TSH, thyroid-stimulating hormone; BMI, body mass index; IQ, intelligence quotient; β, standardized coefficient beta. *, statistically significant association.

## Data Availability

The data that support the findings of this study are available from the corresponding author upon reasonable request.
